# Decision-making in Surgery or Active Surveillance for Low Risk Papillary Thyroid Cancer During the COVID-19 Pandemic

**DOI:** 10.3390/cancers13030371

**Published:** 2021-01-20

**Authors:** Anna M. Sawka, Sangeet Ghai, Ogemdi Ihekire, Jennifer M. Jones, Amiram Gafni, Nancy N. Baxter, David P. Goldstein, on behalf of the Canadian Thyroid Cancer Active Surveillance Study Group

**Affiliations:** 1 Division of Endocrinology, University Health Network and University of Toronto, Toronto, ON M5G 2C4, Canada; 2 Joint Department of Medical Imaging, University Health Network-Mt Sinai Hospital-Women’s College Hospital, University of Toronto, Toronto, ON M5G 2C4, Canada; sangeet.ghai@uhn.ca; 3 Research Associate, Department of Otolaryngology-Head and Neck Surgery, University Health Network, Toronto, ON M5G 2C4, Canada; ogemdi.ihekire@uhn.ca; 4 Department of Psychosocial Oncology, University Health Network and University of Toronto, Toronto, ON M5G 2C4, Canada; jennifer.jones@uhn.ca; 5 Centre for Health Economics and Policy Analysis, Department of Health Research Methods, Evaluation and Implementation, McMaster University, Hamilton, ON L8S 4L8, Canada; gafni@mcmaster.ca; 6 Melbourne School of Population and Global Health, University of Melbourne, Melbourne, VIC 3010, Australia; nancy.baxter@unimelb.edu.au; 7 Princess Margaret Cancer Centre, Department of Otolaryngology-Head and Neck Surgery/Surgical Oncology, University Health Network and University of Toronto, Toronto, ON M5G 2C4, Canada; david.goldstein@uhn.ca

**Keywords:** papillary thyroid cancer 1, active surveillance 2, COVID-19 pandemic 3, SARS-CoV-2 4, virtual care 5

## Abstract

**Simple Summary:**

In March of 2020, the World Health Organization declared a COVID-19 pandemic, which had dramatic implications for thyroid cancer clinical care and research. Beginning early in the pandemic, at the University Health Network in Toronto, cancer care rapidly transitioned from in-person to virtual outpatient cancer care. Elective surgeries were also restricted, particularly for low risk malignancies. We herein discuss our experience conducting an ongoing study on decision-making regarding surgery or active surveillance for small, low risk papillary thyroid cancer (PTC) during the COVID-19 pandemic. Our study protocol was adapted due to safety considerations, including adopting virtual telephone/video teleconferencing patient visits and verbal consent procedures, and allowing for increased flexibility in appointment scheduling. We discuss some preliminary observations on our study process and outcomes during the pandemic.

**Abstract:**

We describe our experience conducting a prospective observational cohort study on the management of small, low risk papillary thyroid cancer during the COVID-19 pandemic. Our study participants are given the choice of active surveillance (AS) or surgery, and those in the AS arm are followed at the study center, whereas surgical patients undergo usual care. During the pandemic we have transitioned from in-person research patient visits to largely virtual care of patients under AS. As of 30 October 2020, we had enrolled 181 patients enrolled in our study (including 25 during the pandemic), of which 92.3% (167/181) consented to telephone communication and 79.0% (143/181) consented to secure videoconferencing communication. Prior to the pandemic, 74.5% (117/157) of our patients chose AS over surgery, whereas during the pandemic, 96.0% (24/25) chose AS. Of the 133 study patients who were under AS within the timeframe from 12 March 2020, to 30 October 2020, the percentage of patients who missed appointments was 8.3% (11/133, for neck ultrasound and physician visits, respectively) and delayed appointments was 23.3% (31/133). This preliminary data suggests that prospective observational research on AS of thyroid cancer can safely continue during the pandemic.

## 1. Introduction

On 11 March 2020, the World Health Organization declared a global pandemic of the novel corona virus, COVID-19 [1]. Consequently, global health services delivery, including provision of cancer care, was dramatically impacted. Important considerations taken in developing health policies during the COVID-19 pandemic include strategies intended to mitigate the risk of viral exposure (and related complications or death) in patients and healthcare workers, as well as the preservation of sufficient inpatient healthcare capacity to care for severely ill infected patients (e.g., healthcare staff, personal protective equipment, inpatient/intensive care unit beds, and intensive care equipment (particularly ventilators)). Studies from various countries have suggested that thyroid cancer care has been significantly impacted by the COVID-19 pandemic, including reports of reductions in: fine needle aspiration biopsies of thyroid nodules [2,3], outpatient visits [3], thyroid surgery volumes [2], and radioactive iodine ablation [3]. Furthermore, virtual care has been increasingly adopted in thyroid cancer outpatient care [4]. Virtual care may be defined as “any interaction occurring remotely between patients and/or members of their circle of care, through any form of communication or information technology with the aim of facilitating or maximizing the quality and effectiveness of patient care” [5]. Some examples of mechanisms that may be used to provide virtual care include telephone, personal videoconferencing, e-mail, or secure messaging.

Ontario is the most populated province in Canada and has been subject to local epidemic spread of COVID-19 since late February of 2020 [6]. On 10 March 2020, Cancer Care Ontario released a clinical practice guideline on pandemic planning for care of patients with cancer [7]. In these guidelines, some key recommendations regarding the provision of outpatient cancer care included the establishment of mechanisms for virtual outpatient visits and deferring (in-person) clinical visits or imaging in asymptomatic patients without life-threatening issues [7]. An important element of the Cancer Care Ontario guidelines was the description of a prioritization scheme for performing surgical procedures during a pandemic [7]. In the Cancer Care Ontario guidelines, well-differentiated thyroid cancer was categorized as being an example of an “indolent tumour”, which was considered of the lowest surgical priority, specifically Priority “C”, assuming that “a delay of 2 months would be unlikely to affect outcome” [7]. Thus, the delay of low risk cancer surgeries, such as thyroid cancer, was an expected outcome in oncologic pandemic planning. In keeping with Cancer Care Ontario guidance, the Ontario Ministry of Health (which is a publicly-funded healthcare funder) directed hospitals to begin “ramping down of elective surgeries and other nonemergent clinical activity” as of March 15, 2020 [8]. As such, in April of 2020, there were 38% fewer cancer surgeries performed in of Ontario, as compared to the same time in the preceding year [8].

The Cancer Care Ontario pandemic planning guidelines were implemented in many Ontario hospitals, including the University Health Network, in Toronto, from the early days of the pandemic. In response to provincial government directives, the University Health Network’s Princess Margaret Cancer Centre executive leadership instructed cancer care providers to aim at reducing in-person ambulatory clinic visits by 50% [9]. Furthermore, elective surgeries for low risk cancer were delayed in our institution, with some resultant backlog in cases.

Since May of 2016, at University Health Network, we have been conducting a study on patient choice and outcomes of surgery or active surveillance (AS) for the management of small, low-risk papillary thyroid cancer (PTC) [10]. In this study, we include patients with thyroid nodules that are either positive or suspicious for papillary thyroid cancer on fine needle aspiration biopsy, where the tumor is <2 cm in maximal diameter, confined to the thyroid (i.e., no known metastatic disease nor extrathyroidal extension), and not in a location that is thought to threaten critical structures (e.g., the trachea or recurrent laryngeal nerve) [10]. In this study, patients who chose AS are followed by the study investigators at the University Health Network, whereas patients who choose surgery receive usual care and follow-up from their treating physician at an institution of their choice [10]. For surgical patients, medical records are reviewed, but there are no study-specific medical investigations or visits. We recently published some preliminary data regarding the rationale for patients’ disease management choices in this study [11]. However, participant recruitment and follow-up for this study is ongoing. The COVID-19 pandemic, and resultant clinical practice changes at the University Health Network, presented new challenges in conducting our study. Our primary considerations in continuing our study have been a focus on patient and staff safety, as well as strict attention to maintaining study integrity. We herein discuss our experience in addressing pandemic-related challenges in conducting our study, as well as associated data on follow-up, and newly recruited patients’ disease management choices.

## 2. Results

### 2.1. Description of the Study Population

**Figure 1:**
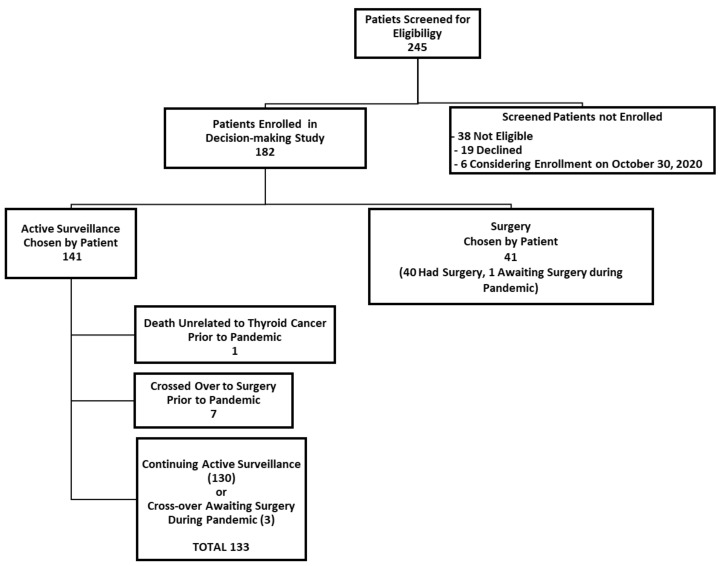
Participant flow diagram.

We herein present some preliminary findings, as of 30 October 2020. At this point, about seven months after the pandemic had been declared, 182 participants had enrolled in our active surveillance or surgery decision-making study. As of this date, 141 patients had chosen to undergo active surveillance (77.5%) and 41 patients chose to have surgery (22.5%). A participant flow diagram is shown in Figure 1.

The demographic characteristics of study participants are shown in Table 1. The mean age of the 182 enrolled study population was 52.0 years (standard deviation, SD 15.1 years), of whom 140 were female (76.9%) and 42 were male (23.1%). The mean duration of follow-up since study enrollment is 22.2 months (standard deviation 14.1 months, minimum 0.2 months and maximum 53.1 months, n = 182). The mean primary tumor size at enrollment was 1.1 cm (SD 0.4 cm, minimum 0.3 cm, maximum 1.9 cm, n = 182). The cytopathology of the primary tumor was papillary thyroid cancer in 70.9% (129/182) of patients and suspicious for papillary thyroid cancer in 29.1% (53/182) of patients.

Of the 141 patients enrolled in our study who chose active surveillance, 130 were continuing active surveillance as of 30 October 2020 (Figure 1). Those who enrolled initially in the active surveillance that were no longer undergoing active surveillance included: one elderly patient who died of unrelated causes (within six months of enrolling in the study, prior to the pandemic) and seven patients who had crossed over to surgery (and completed their surgery) prior to the pandemic (i.e., prior to 11 March 2020). Of the seven patients who initially enrolled in active surveillance but crossed over to surgery prior to the pandemic, the surgical indication was patient choice for six patients and a recommendation of the study investigators in one patient (due to irregularity of the interface of the tumor and thyroid parenchyma (from hashimoto’s thyroiditis), which interfered with the accurate measurement of the tumor in the follow-up). There were three patients in the active surveillance group who crossed over from active surveillance immediately before or during the pandemic, so they underwent active surveillance for at least part of the time during the pandemic (two crossed over to surgery due to patient choice (one awaiting surgery as of 30 October 2020), and one experienced tumor enlargement (awaiting surgery as of 30 October 2020). Thus, a total of 181 study participants from both study arms had been enrolled in the study as of 30 October 2020, and of these, 133 patients underwent active surveillance during at least some portion of the pandemic (from 11 March 2020, until 30 October 2020).

### 2.2. Participant Acceptance of Virtual Communication during the COVID-19 Pandemic

Beginning in 30 April 2020, we contacted all study participants to inform them about the institutional research ethics board approved study procedural changes during the pandemic and answer any potential questions or concerns of participants. Patients were mailed a letter describing the study procedural changes and this was followed up with a phone call from a research assistant, who answered questions and inquired about consent for virtual care.

Patients were asked for verbal consent regarding various aspects of virtual care during the pandemic. Of the 181 patients enrolled in the study, 92.3% (167/181) consented to telephone communication with study staff and doctors, 79.0% (143/181) consented to secure videoconferencing communication with study doctors using the Ontario Telemedicine Network (OTN), and 92.8% (168/181) consented to e-mail communication regarding study appointments and electronic mailing of non-identifiable study materials (such as questionnaires or other documents). We also inquired about whether patients had access to their University Health Network electronic medical record, which information on appointments, diagnostic test results, and physician transcripts of notes from clinical visits. The majority of our study participants (85.6%, 155/181) already had access to their institutional electronic medical record, and the remainders were offered for such access (if interested). Of the 133 patients who underwent active surveillance during at least part of the pandemic, 97.0% (129/133) provided consent for telephone communication, 85.7% provided consent for communication with study doctors through the OTN (114/133), and 96.2% (128/133) provided consent for email communication with study staff for administrative purposes. The majority (88.7%, 118/133) of patients under active surveillance during the pandemic also already had access to their electronic medical records and many patients regularly checked their electronic medical record prior to visits with physicians. Of the four patients under AS who declined any virtual communication consent, one patient later changed that decision and consented (as the patient originally thought the pandemic would be over soon) and the three other study patients had in person visits with the doctor (as per their preference). Patients who had undergone surgery were under usual care and follow-up by their treating physicians and their clinical care would not have been impacted by virtual communication consent.

### 2.3. Impact of the COVID-19 Pandemic on Active Surveillance Follow-Up Procedures

Of the 133 patients who were under active surveillance within the timeframe from 12 March 2020, to 30 October 2020, 8.3% of patients (11/133) had missed (and not made up) a pre-scheduled study clinical appointment with a study physician, 8.3% (11/133) had missed a pre-scheduled study neck ultrasound, and 16.5% (22/133) had missed a blood test (22/133). As of 30 October 2020, 23.3% (31/133) experienced a delayed (but ultimately completed) physician appointment and neck ultrasound, respectively. None of the active surveillance study participants dropped out of the study, however those who missed appointments expressed a preference to temporarily forego or delay appointments, with the intention of resuming diagnostic testing and follow-up in the future (e.g., when the COVID-19 risk was perceived as lower). Prior to the pandemic, all clinical study outpatient visits with study doctors were conducted in person, however only 6.8% (9/133) of study participants had an in-person clinical or research visit during the pandemic. The remainder of all study physician and staff visits with patients were conducted by telephone during the pandemic. As in the case of traditional in person visits, all physician notes from virtual visits were transcribed into the electronic medical record, which patients could review electronically in their medical record, if they chose to do so.

### 2.4. Patients’ Disease Management Choice during the COVID-19 Pandemic

In our study, patients are provided standardized written and verbal information about the options of surgery or active surveillance. Prior to the pandemic, the information on disease management options was exclusively provided in person by a study doctor. During the pandemic, written information was either provided in person or virtually, per patient preference. If a patient chose to receive this information virtually, the written information was sent to the patient’s e-mail link or as a paper copy in the mail. Electronic or paper mail-outs were followed by a virtual phone visit with a study doctor (once the patient had a chance to review the written information). Of the 25 patients enrolled in the decision-making study during the pandemic (from 14 March 2020, to 30 October 2020), most patients were provided disease management information virtually from a study doctor (76.0%, 19/25).

One of the primary aims in our study has been to evaluate how often patients with small, low risk papillary thyroid cancer choose surgery or active surveillance, respectively, as an initial disease management option. As previously reported, 71% (71/100) of the first 100 patients enrolled in our study chose to undergo active surveillance [11]. Of the next 57 patient participants recruited prior to the pandemic, 80.7% (46/57) chose to undergo active surveillance. Thus, in total, prior to the pandemic, 74.5% (117/157) of patients chose active surveillance in lieu of immediate surgery. During the pandemic (14 March 2020 to 30 October 2020), 96.0% (24) of the 25 newly enrolled patients chose active surveillance. All patients were all asked about their rationale for choosing active surveillance or surgery [11]. Regarding patients recruited during the COVID-19 pandemic, none of the patients specifically mentioned “COVID-19” or the “pandemic” in explaining their disease management choice. Furthermore, 64% (16/25) of patients recruited during the pandemic indicated that the disease management choice was made exclusively by them and the remaining 36% (9/25) indicated that the decision was shared between them and their doctor. Thus, all of these patients participated in the decision-making regarding initial management of their thyroid cancer.

## 3. Discussion

The COVID-19 pandemic has transformed cancer care delivery. Furthermore, the COVID-19 pandemic has likely slowed scientific progress in oncologic research [12]. Challenges faced conducting clinical cancer research in studies such as ours include managing reductions in patient recruitment and delayed or missed patient participant appointments, with increased administrative tasks in tracking and evaluating any potential protocol deviations [12]. In our study of low risk papillary thyroid cancer patients, we successfully transitioned from largely in-person to virtual clinical research patient encounters during the pandemic. Telephone was the most popular virtual medium and the participant consent rate for acceptance of clinical study phone calls was high (92.3%, 167/182). Virtual care has been accepted by many Canadian patients. In a recent survey commissioned by the Canadian Medical Association, 34% of 1800 Canadian respondents reported having at least one telephone visit with a doctor during the pandemic and of those, 91% reported being satisfied with the experience [13]. Furthermore, looking to the future (beyond the pandemic), 38% of respondents in the Canadian Medical Association Survey indicated that they would choose the option of telephone, videoconference, e-mail, or text, in lieu of an in-person visit, to access physician medical advice [13]. The follow-up preferences of our study participants are important to examine in the future, as the COVID-19 risk decreases.

Virtual patient care presents both advantages and challenges in the delivery of outpatient healthcare services. Some advantages of virtual care for patients may include: reduced time and expense traveling to appointments, less time missed from work or school, reduced out of pocket expenditures for hospital parking or childcare, and less time spent in hospital waiting rooms [14]. For frail, elderly patients, virtual care may also reduce the challenges of traveling to the hospital and associated caregiver stress and expense. Virtual care may also enable patients to have other family members participate in discussions with them and their physician [15]. Moreover, virtual care may enable patients to have the “time and space” to make medical decisions when they are ready, in their own environment [15], thereby potentially reducing some stress in the medical encounter. However, virtual care may present some challenges in communicating with patients, particularly for individuals who may be hearing impaired or do not speak the language used by the physician [14]. However, the use of personal protective equipment and physical distancing in in-person clinical settings may also create its own challenges in communicating with patients during a pandemic. Pre-scheduling virtual physician visits coordinated with virtual interpreter services (by telephone or videoconferencing) may be helpful for individuals who may require interpretation (e.g., sign language or another language) [14]. As in the case of in-person care, patient privacy and confidentiality must be safeguarded. Privacy considerations in a virtual care context relate to the patient, the provider, and the platform, such that both the patient and physician should be in respective private settings (e.g., room with closed door, away from others that are not participating in the call) and the platform used must be secure (e.g., hospital telephone, institutionally approved videoconferencing platform, or other) [15]. An important concern regarding virtual patient cancer care is that the absence of face-to-face patient-physician communication may impair conveying physician empathy and establishing an emotional connection with the patient, thereby potentially impairing physician trust [14,15,16,17,18]. Furthermore, in the case of thyroid cancer care, virtual care limits the physician’s ability to perform a complete physical examination, particularly palpation of the thyroid and regional lymph nodes. It is important to acknowledge that for some patients in our study, particularly some of those who had recently been diagnosed with thyroid cancer and were considering disease management choices, the opportunity to meet face to face in consultation with a study doctor was important to provide.

An interesting preliminary finding in our study was an observed high rate of acceptance of active surveillance (96.0%, 24/25) as the initial disease management option for patients enrolled during the pandemic. However, patients in the study did not identify the COVID-19 pandemic as the primary reason for their choice. These data are certainly limited by the small number of observations over a relatively short period of time. However, rates of acceptance of active surveillance at our institution were already observed to be increasing prior to the pandemic, in spite of the provision of standardized information on the choice throughout the entire study. Prior to the pandemic, Ito et al. were the first to report on a trend for the increasing rate of acceptance of active surveillance for the management of papillary microcarcinoma in Kuma Hospital in Japan over the preceeding decades [19]. Ito et al. also suggested that “as evidence of the safety and superiority of AS over immediate surgery continues to accumulate, it is expected that the acceptance of this management option will occur more quickly and smoothly in other countries” [19]. Sugitani et al. recently reported that more than half of low risk papillary thyroid microcarcinoma patients are on active surveillance in Japan, based on data from a multi-institutional survey and review of National Cancer Database cases [20]. In Italy, Molinaro et al. also reported that about half of patients (93/185) who were screened and deemed eligible for active surveillance, agreed to undergo active surveillance as part of a prospective research study [21]. In a study from Latin America, Smulever et al. reported that 24% (34/136) of patients with small, low risk thyroid cancer who were eligible for active surveillance, accepted this approach [22]. Our findings appear to confirm the suggestion of Ito et. al. on growing acceptance of active surveillance for small, low risk papillary thyroid cancer, including in countries outside of Japan [19]. It is possible that referring physicians’ confidence in active surveillance as a disease management strategy for low risk papillary microcarcinoma has grown due to a growing foundation of published evidence supporting this intervention as well as its discussion in the American Thyroid Association guidelines on differentiated thyroid cancer management [23] as well as the recently published American Association of Endocrine Surgeons’ guidelines on surgical management of thyroid disease [24]. It is also possible that the COVID-19 pandemic may have influenced patients’ decisions to avoid surgery and to choose AS, although the patients in our study did not specifically mention “COVID-19” or the “pandemic” in explaining their choices. An important limitation of our study is that we did not perform an in-depth qualitative interview with additional questions probing on the potential impact of the pandemic. Any potential impact of the COVID-19 pandemic on patients’ decision-making on disease management of small, low risk PTC, is currently unclear, but certainly deserves further in-depth study [25].

Prior to the pandemic, authors of studies of active surveillance of small, low risk papillary thyroid cancer reported relatively high rates of continued patient acceptance of active surveillance at long-term follow-up [21,22,26,27,28,29,30]. Davies et al. have recently reported that, in the Kuma Hospital experience, factors enabling continued patient acceptance of active surveillance at follow-up included: receiving detailed test results, patient education (about active surveillance), and supportive interactions with the thyroid cancer physician [26]. It is too early in the follow-up trajectory of patient participants in our study to meaningfully examine long-term retention of patients in the active surveillance arm of the study. However, our preliminary data suggests that even throughout the pandemic, patients have been largely willing to continue follow-up, albeit sometimes with appointment delays. The impact of virtual patient follow-up on adherence with active surveillance procedures and acceptance of continued active surveillance in the context of thyroid cancer surveillance, is unknown and deserves further study. As suggested by Nickel et al., pandemic-associated changes in thyroid cancer care may serve as a research learning opportunity [25] and we hope that sharing our experience may be helpful to others in conducting or planning clinical research in these unprecedented, challenging times.

## 4. Materials and Methods

Our full study protocol was previously published [10] and the study was registered at Clinicaltrials.gov (NCT03271892) [10]. The study included individuals aged 18 years of age or older who have a thyroid nodule that was <2 cm in maximal diameter, with a fine needle aspiration biopsy specimen that was positive or suspicious for PTC, and where the thyroid cancer was confined to the thyroid and not in a critical location (relative to the trachea or recurrent laryngeal nerve) [3]. Individuals must have been surgical candidates and were able to consent and comply with study procedures. Patients are offered standardized written and verbal information about the options of surgery or active surveillance of PTC and the final disease management choice was made by the patient. Patients who chose to undergo surgery by a surgeon of their choice, in an institution of their choice, and were provided usual care by their thyroid cancer specialists (surgeons, endocrinologists, or other physicians). Patients who chose active surveillance were followed by investigators (AMS, DPG) in the study center, which involved thyroid ultrasound, bloodwork (TSH, Free T4, thyroglobulin, thyroglobulin antibody) and physician assessment/discussion of test results every 6 months for the first 2 years of active surveillance, and thereafter, yearly. More frequent tests or appointments or additional tests may be scheduled, if clinically indicated (e.g., concern about possible disease progression). Disease progression prompting a surgical recommendation included primary tumor growth in largest dimension >3 mm compared to baseline (confirmed on 2 consecutive ultrasounds), concern about location of growth (e.g., extrathyroidal extension, risk of invasion of the trachea or recurrent laryngeal nerve), or evidence of metastatic disease (local or distant). Patients were are able to chose to cross over from active surveillance to surgery at any time point in the follow-up, in the absence of disease progression, and continued to be followed for clinical outcomes. The study was initiated in May 2016, and participant recruitment and follow-up are ongoing (sample size target of a total of 200 patients followed for in total in the active surveillance and surgical arms, combined). The study was approved by the University Health Network Research Ethics Board.

On 30 April 2020, the University Health Network Research Ethics Board approved a study amendment to enable safe execution of the study during the COVOID-19 pandemic, which included: a formal procedure for obtaining verbal consents for study participation, acceptability of virtual communication of study staff and doctors with participants (after patient consent was formally obtained for such communication), temporary cancellation or delay of some clinical study visits or diagnostic tests in patients in the active surveillance arm of the study (particularly when the community COVID-19 risk is high for patient participants who are at high risk of COVID-19 complications or death). For clinical appointments and tests of patients under active surveillance, the timing and nature of the delay and cancellation was at the discretion of the study doctors who considered each patient’s individual risk of COVID exposure/complications and thyroid cancer risk, the local community risk of COVID-19 infection (reflected by daily and weekly new case counts and hospitalizations), and the institutional availability of clinical services (e.g., medical imaging). As ultrasound imaging was deemed to be a critically important procedure for the evaluation of clinical study outcomes for patients under active surveillance, study imaging was retained at the participating institution. However, thyroid-related blood tests performed in outside laboratories were deemed acceptable for study purposes, if this was considered more safe or feasible for the patient during the COVID-19 pandemic. For appointments that were rescheduled, these were typically performed in summer and early fall months, when the community risk of COVID-19 was the lowest and personal protective equipment for hospital staff was adequate in supply. Routine baseline laryngoscopy in asymptomatic patients were no longer mandated as part of evaluation for eligibility for study participation, as the risk of patient or staff potential COVID-19 exposure and transmission outweighed the potential benefit for the study. Certainly, laryngoscopy was permissible, if clinically indicated (e.g., question of related symptoms or a tumor that is near the recurrent laryngeal nerve). Institutional infrastructure, which enabled study execution during the COVID-19 pandemic included: remote secure staff access to a research server, remote access to the institutional electronic medical record (by patients and staff), and access to a clinical research unit outside of the hospital setting for any necessary in person visits. All study participants were mailed information about the changes in the study in the context of the COVID-19 pandemic and the mail-out was followed up by a phone call by a research assistant (OI), who answered any questions and obtained verbal consent for future virtual communication (if the patient agreed).

Data on verbal consents (including consent for virtual communication) and any COVID-19 pandemic-related protocol deviations (e.g., missed appointments) were entered by a research assistant (OI) in a Microsoft Excel Spreadsheet. Other study data (including demographic and clinical characteristics as well as questionnaire data) was entered into an online program (DADOS, Techna Institute) and the data were merged according to the participant ID number in one spreadsheet for analysis. The dataset was checked by an investigator for any errors (AMS). Data were reported for the entire study population, as well as a subgroup of patients under active surveillance during the COVID-19 pandemic (i.e., patients enrolled in the active surveillance arm who had not crossed over to surgery or dieted prior to the pandemic). Descriptive statistics were reported, including counts and proportions for categorical variables and means, standard deviations, and minimum and maximum values for continuous variables. The analysis of COVID-related study statistics should be considered post-hoc and hypothesis-generating, as these was not planned at the beginning of the study in 2016.

## 5. Conclusions

In conclusion, in this study examining initial treatment decision-making and long-term outcomes of patients with small, low risk papillary thyroid cancer, we have been successful in continuing to conduct our study throughout the pandemic. This process has been enabled by transitioning from largely in-person study visits (particularly for active surveillance physician follow-up visits), to largely virtual care visits. Furthermore, research ethics board approval for a verbal patient consent process was helpful in maintaining participant study recruitment as well as establishing a record of individualized consents for various aspects of virtual care and study contact. We also believe it is important for some patients, particularly those newly diagnosed with thyroid cancer, to have an opportunity for safe, in-person contact with a study physician, if they prefer. This, of course, requires the ample supply of personal protective equipment and COVID-screening of all study staff and patients. As we navigate through the future, and hopefully a swift end to the pandemic, it will be important for us to continue to study patient preferences and outcomes relating to healthcare delivery, particularly relating to virtual care.

## Figures and Tables

**Table 1:** Clinical and demographic characteristics of the 182 study participants.

Characteristic	Number (Percentage) or Mean (Standard Deviation [SD], Range)
Study Arm	141 Active Surveillance (77.5%), 41 Surgery (22.5%)
Sex	140 Females (76.9%), 42 Males (23.1%)
Age (years) ^1^	Mean 52.0 years (SD 15.1, minimum 20, maximum 85)
Follow-up Duration (months)	Mean 22.2 months (SD 14.1, minimum 0.2, maximum 53.1)
Primary tumor size (cm)	Mean 1.1 cm (SD 0.4, minimum 0.3, maximum 1.9)
Cytology of Primary Tumor	129 PTC ^2^ (70.9%), 53 Suspicious for PTC ^2^ (29.1%)

^1^ Age at study enrollment. ^2^ PTC, Papillary Thyroid Cancer.
